# Accelerated microglial pathology is associated with Aβ plaques in mouse models of Alzheimer’s disease

**DOI:** 10.1111/acel.12210

**Published:** 2014-03-18

**Authors:** Rona Baron, Alicia A Babcock, Anna Nemirovsky, Bente Finsen, Alon Monsonego

**Affiliations:** 1 The Shraga Segal Department of Microbiology and Immunology, Faculty of Health Sciences and the National Institute of Biotechnology in the Negev, Ben-Gurion University of the NegevBeer-Sheva, 84105, Israel; 2 Department of Neurobiology Research, Institute of Molecular Medicine, University of Southern DenmarkDK-5000, Odense, Denmark

**Keywords:** aging, Alzheimer’s disease, amyloid beta-peptide, inflammation, microglia, morphology

## Abstract

Microglia integrate within the neural tissue with a distinct ramified morphology through which they scan the surrounding neuronal network. Here, we used a digital tool for the quantitative morphometric characterization of fine cortical microglial structures in mice, and the changes they undergo with aging and in Alzheimer’s-like disease. We show that, compared with microglia in young mice, microglia in old mice are less ramified and possess fewer branches and fine processes along with a slightly increased proinflammatory cytokine expression. A similar microglial pathology appeared 6–12 months earlier in mouse models of Alzheimer’s disease (AD), along with a significant increase in brain parenchyma lacking coverage by microglial processes. We further demonstrate that microglia near amyloid plaques acquire unique activated phenotypes with impaired process complexity. We thus show that along with a chronic proinflammatory reaction in the brain, aging causes a significant reduction in the capacity of microglia to scan their environment. This type of pathology is markedly accelerated in mouse models of AD, resulting in a severe microglial process deficiency, and possibly contributing to enhanced cognitive decline.

## Introduction

Microglia, the immune resident cells of the brain, constantly survey their surrounding for pathogens (Nimmerjahn *et al*., 2005; Wake *et al*., 2009; Tremblay *et al*., 2011). Although traditionally characterized by their role in host defense, a growing body of evidence suggests that microglia also contribute to the integrity and maintenance of the neuronal network (Sierra *et al*., 2010; Tremblay *et al*., 2011; Schafer *et al*., 2012; Kettenmann *et al*., 2013). The frequency and duration of microglia–neuron interactions, for instance, are influenced by local neuronal activity (Wake *et al*., 2009; Tremblay *et al*., 2011), and glutamatergic and GABAergic neurotransmission were shown to affect the dynamics of microglial process motility in the healthy brain (Fontainhas *et al*., 2011). In addition, removal of dendritic spines by microglia was recently demonstrated to contribute to neuronal activity-based synaptic pruning during the postnatal development of the retinogeniculate system. Expression of complement receptor 3 (CR3) by microglia was essential for synaptic pruning in this system (Schafer *et al*., 2012).

Given that the majority of adult microglia stem from yolk-sac macrophages that populate the CNS during development (Saijo & Glass, 2011), and that their turnover by circulating monocytes is very limited, microglia may be subjected to senescence mechanisms that impair their function (Streit & Xue, 2009; Tremblay *et al*., 2012). This raises the question whether, in contrast to neuronal damage induced by microglial activation, age-related neurodegenerative diseases are in fact caused by microglial dysfunction (Hanisch & Kettenmann, 2007; Lucin & Wyss-Coray, 2009; Streit & Xue, 2009). Streit and colleagues (Streit & Xue, 2009) have shown that human microglia stained with the HLA-DR antibody exhibit dystrophic morphology during aging, characterized by fragmentation, loss of fine processes, and swellings at the terminals of cytoplasmic processes (Streit & Xue, 2009). In addition, compared with retinal microglia in young mice, aged microglia showed significantly smaller and fewer branched processes, slower process formation and motility, and a poor response to laser-induced focal tissue injury (Damani *et al*., 2011). It has also been argued that microglia showing an activated-like morphology, such as those around Aβ plaques in brains affected with Alzheimer’s disease (AD), are in fact maintained quiescent unless peripheral inflammation ensues (Perry *et al*., 2003; Cardona *et al*., 2006; Hickman *et al*., 2008; Streit & Xue, 2009; Abutbul *et al*., 2012).

In light of this accumulating body of evidence, establishing a precise morphological analysis of microglia in the adult brain, and the changes they undergo during aging and disease, may reveal key aspects of neurodegeneration and cognitive impairment. In this study, we use morphometric and FACS analyses to quantitatively define alterations in the fine structure of microglia during aging and in AD-like disease.

## Results

### Quantitative analysis of microglial morphology

**Figure 1:**
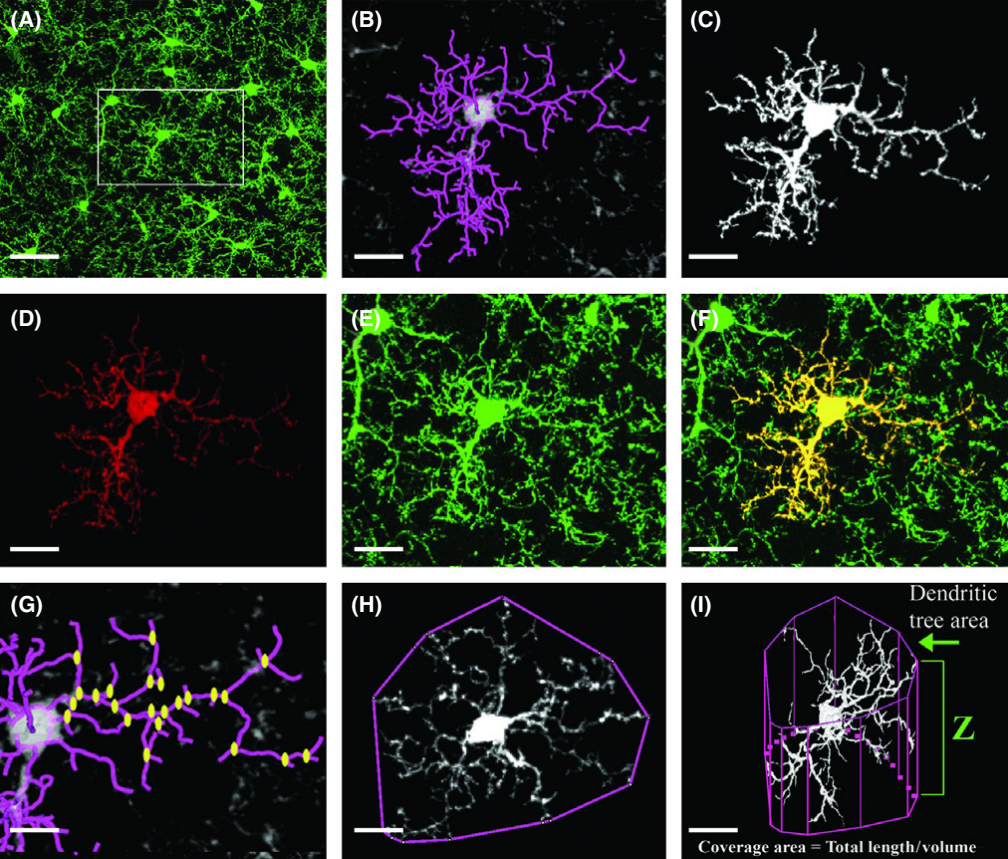
3-D morphological analysis of individual microglia. Brains from young (3 months old) CX3CR1^GFP^^/+^ Tg mice were fixed, sectioned, and subjected to confocal microscopy and digital image analysis as described in Experimental procedures. (A) A representative z-projection image of GFP-labeled cells, showing the distribution of microglia in the cortex. Framed area is enlarged in (B–F). (B) The backbone (purple-colored processes) of a single cell traced with the Simple Neurite Tracer plug-in. (C) A 3-D view of the single cell generated with the ‘filling option’ within Simple Neurite Tracer plug-in and exported for visualization with the Volocity software. (D) A z-projection of the cell, overlaid on top of the original z-projection GFP image (E, enlargement of the framed area in A). (F) The merged image of D and E, showing the individual microglia cell analyzed in yellow. (G–I) The principle parameters analyzed with the L-measure software: (G) the total number of bifurcations (yellow dots) and the total branch length (purple-colored processes); (H) the tree area, calculated as the area constrained by the polygonal object defined by connecting the outer points of the branches; (I) the coverage volume of individual microglia, calculated as the total branch length of the cell divided by its volume and normalized for a 10 μm^3^ unit of tissue volume. The volume of microglia was calculated by the total tree area multiplied by the cell depth (Z). Bars represent 20 μm (A, I); 15 μm (B–F, H); 10 μm (G).

Microglia in the adult mouse brain exhibit ramified morphologies that differ in cell density and process ramification at distinct anatomical regions of the CNS (Olah *et al*., 2011). To characterize the morphological changes microglia undergo with age and disease, we prepared sagittal brain sections from heterozygous CX3CR1 GFP knock-in mice (CX3CR1^GFP/+^), imaged the microglia at layers 2/3 of the cortex (0 to −2 mm lateral to bregma) using a confocal microscope, and digitally analyzed the data as described in Experimental procedures. Figure 1A demonstrates that the entire microglial population in the brain is GFP positive, forming an extensive network of processes. Given the constitutively high levels of CX3CR1 in microglia (Cardona *et al*., 2006), the cells are normally GFP-bright (Fig. 1A), which allows analysis of process arborization of individual cells. We thus took serial z-stack images (every 0.5 μm throughout 50-μm-thick brain sections) and manually traced the backbone of the cells throughout the z-stack images with the FIJI software (Schindelin *et al*., 2012) and the Simple Neurite Tracer plug-in (Longair *et al*., 2011), as described in Experimental procedures. Figure 1B is a representative image of the backbone of one distinct microglial cell within the tissue. Based on this tracing, we then used the filling option of the plug-in to reconstruct the fine processes of the cell (Fig. 1C). Overlaying a z-projection of the reconstructed cell (Fig. 1D) on top of the original z-projection GFP image (Fig. 1E), yielded a precise overlay in yellow (Fig. 1F), which reliably distinguishes processes of the analyzed cell from those of neighboring cells. The data obtained with the plug-in were further analyzed with the L-measure software (Scorcioni *et al*., 2008) to extract the following parameters: (i) total branch length, namely the sum of all branch segments identified with the Simple Neurite Tracer plug-in; (ii) number of branches and bifurcations (Fig. 1G); (iii) total tree area measured on the z-projection images by encircling the edge points of all branches (Fig. 1H); and (iv) coverage volume of individual microglia, which was calculated as the total branch length divided by microglial volume (Fig. 1I).

### Microglial morphology is altered in the cortex of old mice

**Figure 2:**
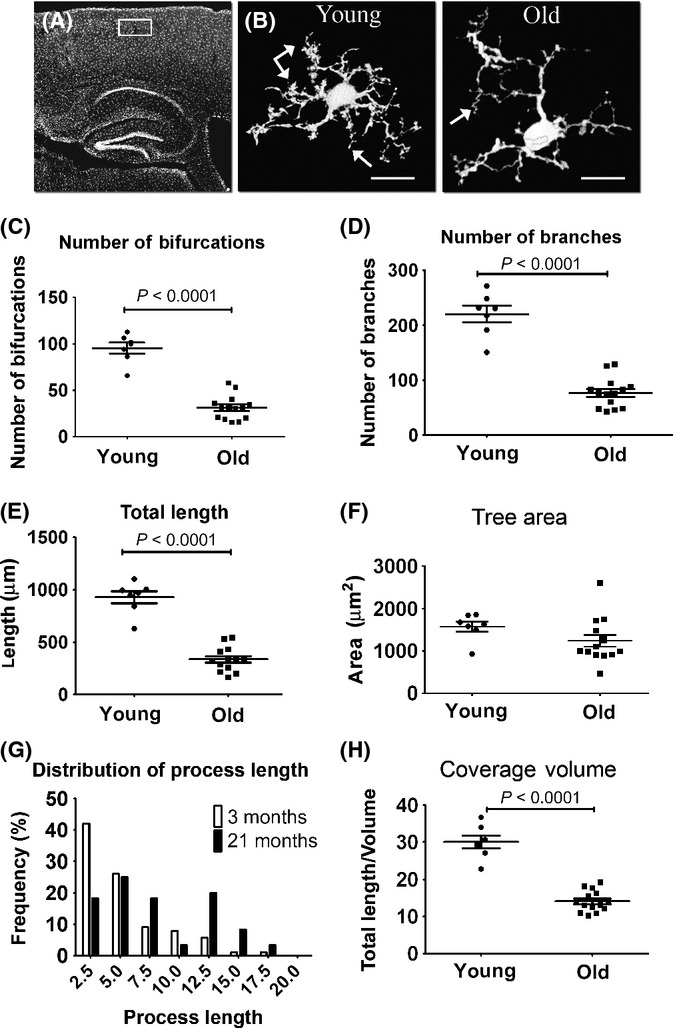
GFP-labeled microglial process complexity deteriorates with aging. Young (3 months old) and old (21 months old) CX3CR1^+/^^GFP^ Tg mice were killed, and brain sections were quantitatively analyzed for individual microglial morphology. (A) Overview image of a sagittal brain section showing the cortical area (area denoted by a white rectangle) used for morphological analysis. (B) Representative z-projection images of microglia from young (left panel, arrows point to fine tips) and old (right panel, arrows show twisted processes) mice, traced with the Simple Neurite Tracer plug-in. (C–F) Morphological analysis of randomly selected young and old cortical microglia (layer 2/3), preformed with the L-measure software and statistically analyzed with GraphPad. Scatter plot graphs show means (horizontal line) ±SEM, and each dot represents one cell analyzed. Graphs indicate the number of bifurcations (C), the number of branches (D), the total branch length (E), and the total tree area (F) of individual microglia in young (three mice; *n* = 7 cells) and old (five mice; *n* = 14 cells) mice. (G) A histogram indicating the distribution of microglial process length. (H) The coverage volume governed by individual microglia cells, depicted by the length of processes in a 10 μm^3^ unit of tissue. *P* values were calculated with Student’s *t*-test. See Experimental procedures for further details. Bar represents 15 μm (young), 10 μm (old).

Having established the ability to trace individual microglial cells, we continued to quantitatively analyze microglial processes in layer 2–3 of the cortex (Fig. 2A) of young (3 months old) and old (21 months old) mice. Representative cells extracted with the Simple Neurite Tracer plug-in are shown in Fig. 2B. Cells extracted from old mice had fewer processes, mostly fine processes at the tip of the branches, than cells extracted from young mice (Fig. 2B). In addition, some abnormal twisted processes were observed in the old mice (Fig. 2B). Quantitative analysis of microglial processes revealed, on average, fewer bifurcations (*P* < 0.0001), fewer branches (*P* < 0.0001), and reduced total branch length (*P* < 0.0001) in old compared with young mice (Fig. 2C–E and Table S1, Supporting information). Although a significant decrease in arborization parameters was observed, the total tree area was slightly but not significantly reduced, namely from 1574 ± 119 to 1240 ± 140 μm^2^ (mean ± SD) in young vs. old mice, respectively (Fig. 2F and Table S1). A branch-length distribution histogram revealed that microglia from old mice also possess fewer short branches (average 2.5 μm) and more long processes (> 5 μm) than microglia from young mice (Fig. 2G). We also calculated the length of branches within a 10 μm^3^ unit of tissue volume by dividing the total branch length of an individual microglia by its volume (calculated by multiplying the tree area parameter by the depth of the microglia, as revealed by the tracing analysis). Notably, this analysis revealed that microglial coverage volume is reduced by more than 50% in old mice, compared with young mice (*P* < 0.0001, Fig. 2H and Table S1). Thus, considering the dynamic nature of the microglial processes, such a robust loss of branches during aging may significantly impair the overall sensing capacity of microglia.

As IbaI is commonly used for morphometric and ultrastructural analyses of microglia (Shapiro *et al*., 2009), we performed the same morphological analysis in brain sections immunolabeled with anti-IbaI (Fig. S1). Overall, similar to our findings in CX3CR1^GFP/+^ mice, the analysis of microglial morphology based on IbaI expression in WT mice revealed a significant reduction in process formation and microglial volume coverage in old mice (Fig. S1).

### Expression of proinflammatory cytokines is mildly increased with aging

It is well known that the levels of proinflammatory cytokines of the innate immune system increase in the CNS during aging (Perry *et al*., 2003; Lucin & Wyss-Coray, 2009). We therefore sought to determine whether the reduction in volume coverage by microglial processes observed in old mice coincided with increased expression of proinflammatory cytokines. Brain tissues from young, adult, and old mice were thus subjected to quantitative PCR analysis of TNF-α, IL-1β, and IL-6. Except for IL-6, which first became upregulated in adult mice, a two- to threefold upregulation of the proinflammatory cytokines was evident in brains of aged mice (Table 1). These data suggest that during the process of aging, microglia exhibit a proinflammatory profile plausibly underlying their reduced morphological complexity.

**Table 1:** Expression of proinflammatory cytokines in adult and old mice

Cytokine	Adult (fold change ± SD)	Old (fold change ± SD)
IL-1β	1.45 ± 0.21	3.28 ± 0.83 (*P* < 0.01)
IL-6	1.83 ± 0.2 (*P* < 0.01)	2.34 ± 0.13 (*P* < 0.001)
TNF-α	0.96 ± 0.1	3.07 ± 0.75 (*P* < 0.01)

Young (aged 6–8 weeks, *n* = 3), adult (aged 7–8 months, *n* = 4), and old (aged 19 months, *n* = 6) C57BL6 mice were analyzed for IL-1β, IL-6, and TNFα gene expression in the brain as described in Experimental procedures. Data are presented as fold change compared with young mice. Data were analyzed by a one-way Tukey’s ANOVA.

### Microglial morphology is altered in the cortex of APP Tg mice

One of the hallmarks of AD pathology is the accumulation of Aβ species, generated by alternative cleavage of the amyloid precursor protein (APP), in diffuse and condensed plaques in the brain (Hardy & Selkoe, 2002; Eisenberg & Jucker, 2012). Plaques are typically observed, in the brain of patients with AD as well as in brains of murine models of the disease, mainly in the hippocampus and cortex (Hardy & Selkoe, 2002). Both the soluble and aggregate forms of Aβ are toxic and may induce glial activation and neuronal loss (Benilova *et al*., 2012; Eisenberg & Jucker, 2012). Indeed, microglia are more abundant at sites of Aβ plaques, with altered morphology reminiscent of cell activation (Mucke *et al*., 2000; Hickman *et al*., 2008; Eisenberg & Jucker, 2012).

**Figure 3:**
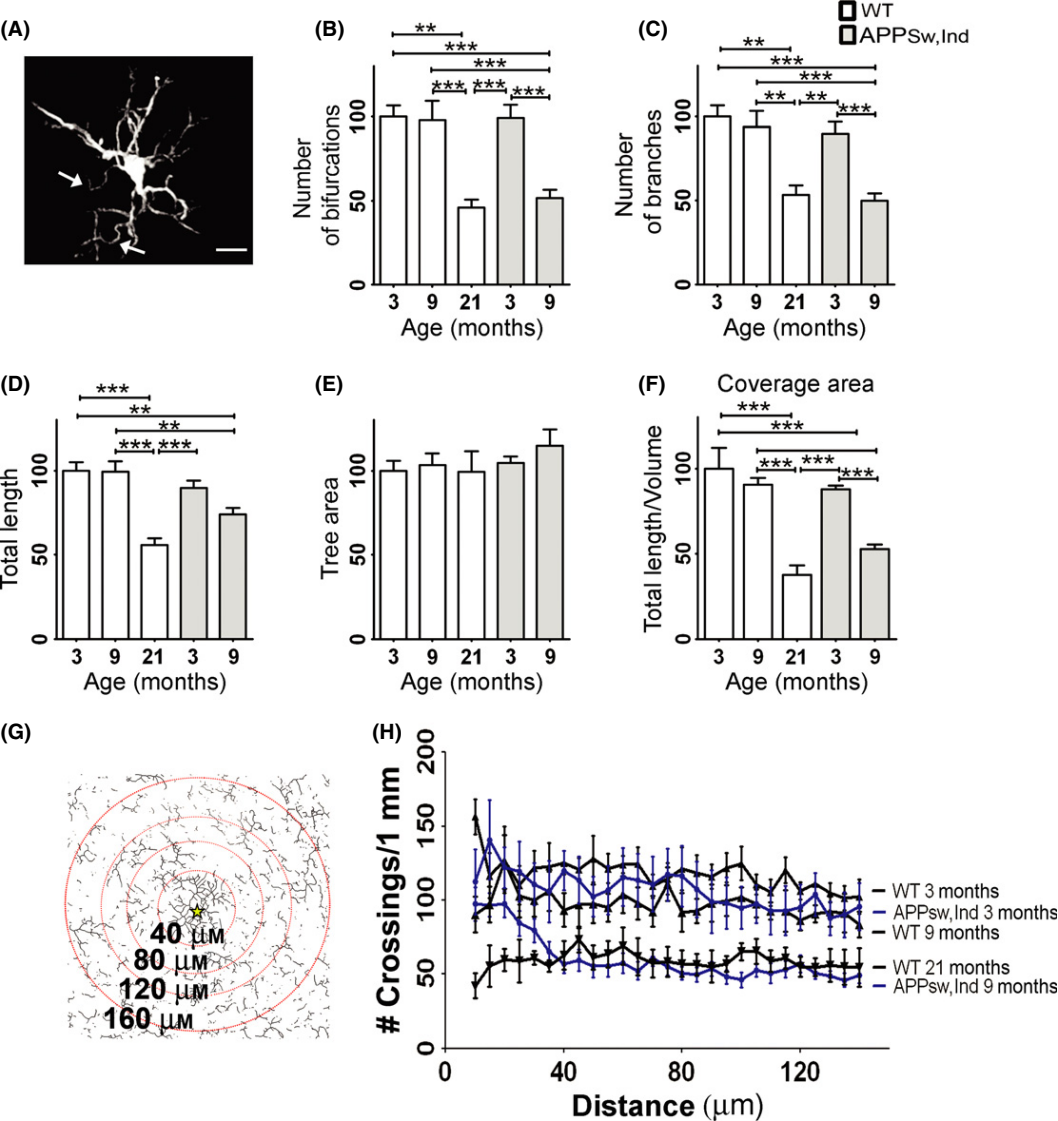
Microglial morphology is altered in a mouse model of Alzheimer’s disease (AD). Brains from young (3 months old) and adult (9 months old) APP_S__w,Ind_ Tg mice were immnuolabeled with IbaI and quantitatively analyzed for individual microglial morphology compared with 3-, 9-, and 21-month-old WT mice. (A) A representative image of IbaI+ microglial cell from a 9-month-old APP_S__w,Ind_ Tg mouse, generated by the Simple Neurite Tracer plug-in. Arrows indicate aberrant twisted processes. (B–F) Morphometric parameters were analyzed with the L-measure software based on IbaI staining in 3- (three mice; *n* = 15) and 9-month-old (three mice; *n* = 10) APP_Sw,Ind_ Tg mice and WT mice aged 3 (three mice; *n* = 10 cells), 9 (five mice, *n* = 14 cells) and 21 (three mice; *n* = 8 cells) months. To compare the groups, data were first normalized to 3-month-old WT mice. Graphs show means of percent change ± SEM of the number (#) of bifurcations (B), number of branches (C), total branch length (D), tree area (E), and volume coverage of individual microglia cells (F). (G-H) Sholl’s analysis indicating the number of microglial processes crossing concentric circles drawn at 5-μm intervals around Aβ plaques (a star marks the plaque center) in APP_Sw,Ind_ Tg mice, or an arbitrary location in the cortex of WT mice (aged 3, 9, and 21 months). A representative illustration of the modified Sholl’s analysis for APP_Sw,Ind_ Tg mice is shown in G. For clarity, only a fraction of the circles is shown. Data were analyzed by a one-way Tukey’s ANOVA. *P < 0.05; **P < 0.01, ***P < 0.001. Bar represents 10 μm.

To test whether microglial morphology is altered due to Aβ accumulation, we analyzed two mouse models of AD that differ in the distribution of Aβ plaques in the brain; whereas Aβ accumulates more abundantly in the hippocampus in APP_Sw,Ind_ Tg mice (Mucke *et al*., 2000), the Aβ plaques are evenly distributed in the hippocampus and cortex in APP/PS1 Tg mice (Jankowsky *et al*., 2004) (Fig. S2, upper panels). In both models, microglia are clustered around Aβ plaques, leaving the surrounding tissue covered by fewer processes than usually occurs in age-matched WT mice (Fig. S2, lower panels). We first examined how the morphology of individual microglia is altered in adult APP_Sw,Ind_ Tg mice (9 months old) due to the low density of Aβ plaques in their cortex. Brain sections from young and adult APP_Sw,Ind_ Tg mice were thus immunolabeled with IbaI, and microglial morphology at plaque-free areas in the neocortex was compared with those in young, adult, and old (3, 9, and 21 months old, respectively) WT mice (Fig. 3). As previously described (Streit & Xue, 2009), microglia from adult APP_Sw,Ind_ Tg mice showed curved and twisted processes (Fig. 3A). To quantitatively compare the groups, we normalized all the parameters with respect to the young WT mice. The comparison revealed a significant reduction in the number of bifurcations and branches, and of the total microglial process length, in adult APP_Sw,Ind_ Tg mice compared with age-matched WT and young APP_Sw,Ind_ Tg mice, resulting in values similar to those observed in old WT mice (Fig. 3B–D). As in the old WT mice, coverage volume by individual microglia cells was significantly reduced in adult (*P* < 0.0001), but not in young, APP_Sw,Ind_ Tg mice by almost 50% compared with age-matched mice, with no change in the tree area parameter (Fig. 3E,F).

To quantify the reduction in microglial spatial coverage in the area surrounding Aβ plaques, we used a modified Sholl’s analysis (Fig. 3G,H). Sholl’s analysis was tailored to calculate the number of intersections between microglial processes and concentric circles around Aβ plaques or in randomly selected images taken from the same cortical area of WT mice (3, 9, and 21 months old). The number of intersections per 1 mm length in circles with radius between 40 μm (minimal distance from the center of the plaque) and 140 μm (the average distance to the neighboring plaque) was measured with 5-μm intervals (Fig. 3G,H). The average number of crossings was reduced from 118.1 ± 2.1 in young WT mice to an average of 97.2 ± 1.6 in adult WT mice (*P* < 0.0001) and was further reduced to 59.0 ± 1.1 (*P* < 0.0001) in old WT mice. The number of crossing in young APP_Sw,Ind_ Tg mice was slightly reduced to 106 ± 2.4 (*P* < 0.01) compared with age-matched WT mice and was further reduced to 60.4 ± 3.1 in adult APP_Sw,Ind_ Tg mice (*P* < 0.0001) (Fig. 3H).

**Figure 4:**
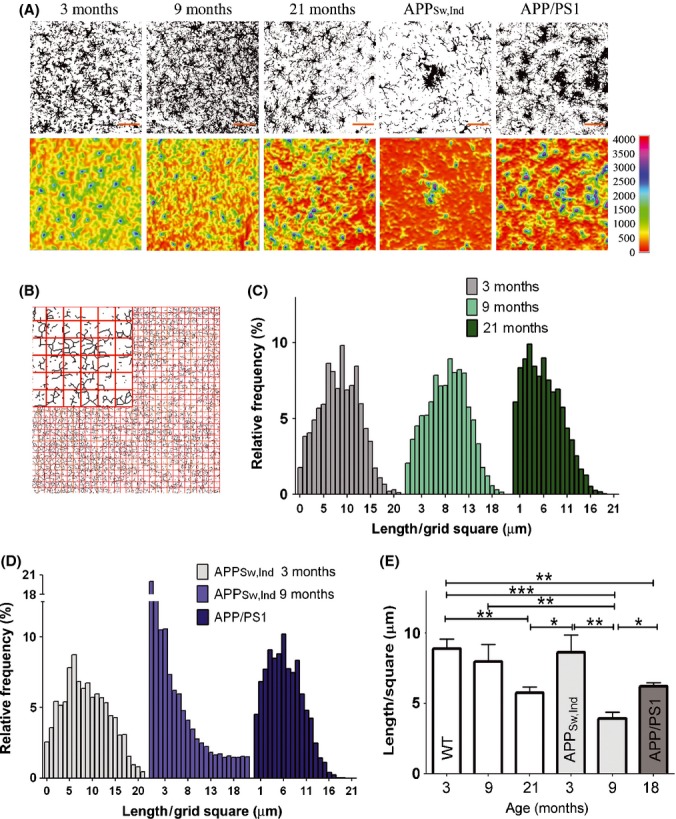
The spatial coverage area of microglia is reduced during aging and in a mouse model of Alzheimer’s disease (AD). Brain sections were taken from WT mice aged 3, 9, and 21 months and from two different AD mouse models, namely the APP_Sw,Ind_ (9 months old) and the amyloid precursor proteins (APP)/PS1 (15 months old) Tg mice, and immunolabeled with anti-IbaI. (A) Confocal z-stack images showing microglia process densities. To classify microglia, gray level, maximum z-projection images were generated and then set to eliminate background based on intensity threshold (upper panels). A heat map visualizing microglia process densities (lower panels) was then generated with the 3D Surface Plot plug-in bundled with FIJI software. Areas highly condensed with microglial processes appear in blue-purple, and areas vacant of microglia processes appear in red-yellow. Bars represent 50 μm. (B–E) Spatial coverage of microglia was performed by grid analysis. Grid analysis was performed by opening a grid of 32*32 squares (each square is 25 μm^2^) on top of a skeletonized image (see a zoom-in image of the grid in panel B). The total calculated length of processes in each square of the grid is presented as a distribution histogram with binning of 1 μm for 3-, 9-, and 21-month-old mice (C) and for APP_Sw,Ind_ and, APP/PS1 Tg mice compared with 3-month-old APP_Sw,Ind_ Tg mice (D). The length of processes per 1 square of a grid was calculated for each mouse (WT, 3 months *n* = 3; all other groups *n* = 5) and averaged for each group. Data were then analyzed by a one-way Tukey’s ANOVA. *P < 0.05; **P < 0.01, ***P < 0.001 (E).

We next examined the changes in spatial microglial coverage at sites of Aβ plaques (Fig. 4). Confocal z-stack images (1-μm intervals over 20-μm-thick brain tissue) were taken from young, adult, and old WT mice and from the two mouse models of AD. Using the FIJI software (see details in Experimental procedures), images were processed as follows: (i) Gray-level maximum z-projection images were generated and then set to eliminate background based on intensity threshold (Fig. 4A, upper row); (ii) 3-D surface plots were generated from the z-stack confocal images; and (iii) images were skeletonized depicting the backbone of the arborized microglial structure (data not shown). Collectively, these images demonstrated that whereas microglia are tightly packed and cover the tissue uniformly in young mice, coverage is impaired in old mice and more severely in 9-month-old APP_Sw,Ind_ Tg mice, leaving tissue space devoid of microglia processes (Fig. 4A). The reduction in microglial spatial coverage in the cortex of 15-month-old APP/PS1 Tg mice was similar to that of old mice and less severe than in APP_Sw,Ind_ Tg mice (Fig. 4A).

To quantify the spatial coverage of microglial processes, we overlaid a grid on top of the skeletonized image (as illustrated in Fig. 4B) and calculated the total length of processes in each cell of the grid. A distribution histogram was then generated based on this grid analysis (Fig. 4C,D), revealing normal distribution around an average length of microglial processes, which was higher in young mice and was gradually reduced with age. The average length of microglial processes in a grid square decreased from 8.87 ± 0.69 μm in 3-month-old mice to 7.96 ± 1.22 and 5.75 ± 0.42 μm (*P* < 0.01, comparison of 3- to 21-month-old groups) in 9- and 21-month-old mice, respectively (Fig. 4E). Compared with 3-month-old APP_Sw,Ind_ Tg mice, the average length in a grid square further decreased from 8.66 ± 1.24 μm to 3.91 ± 0.46 (*P* < 0.001) and 6.21 ± 0.26 in adult APP_Sw,Ind_ and old APP/PS1 Tg mice, respectively (Fig. 4E). Vacant areas, defined by the number of cells in the grid containing < 1 μm length of processes, increased from 1.7% of the total area in young mice to 6% and 4.5% in old WT mice and 15-month-old APP/PS1 Tg mice, respectively (Fig. 4C,D). Notably, the amount of vacant areas in 9-month-old APP_Sw,Ind_ Tg mice was sharply increased to 20% of the total area (Fig. 4D).

### Differential pattern of CD39 expression in plaque-associated microglia

**Figure 5:**
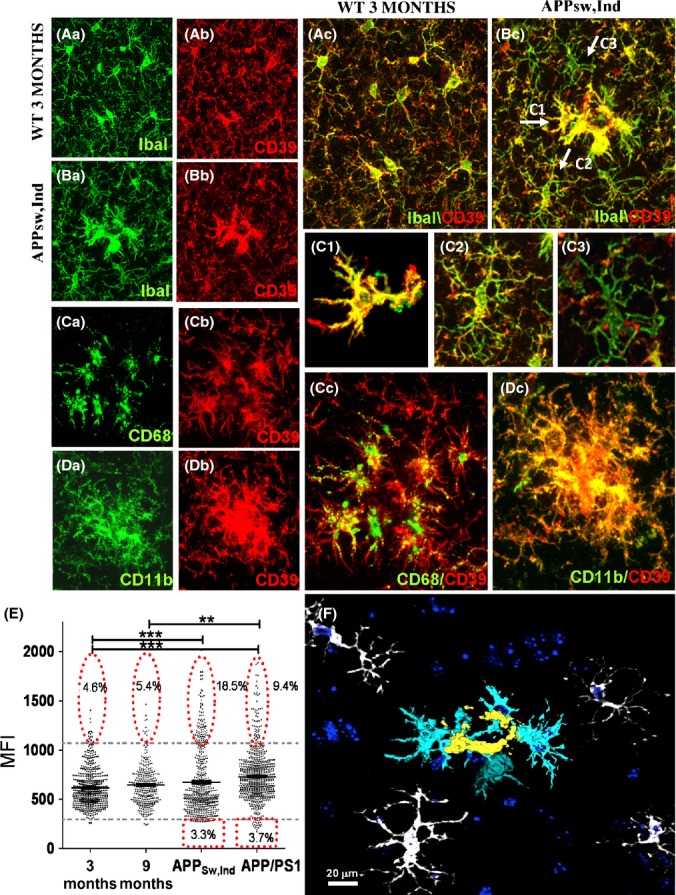
Microglial expression of CD39 is altered in mouse models of Alzheimer’s disease. IbaI and CD39 IHC analyses were performed on brain sections from young, old, and amyloid precursor proteins (APP) Tg mice as described in Experimental procedures. (A, B) Representative z-stack images of WT (aged 3 months) and APP_sw,Ind_ Tg mice (aged 9 months), showing IbaI+ cells (Aa, Ba; green), CD39^+^ cells (Ab, Bb; red), and the merge images (Ac, Bc) in the cortex. Arrows in Bc point to cells classified as CD39^high^ (C1), CD39^+^ (C2), and CD39^low^ (C3). (C1–C3) Enlargement of cells depicted in Bc. Cells shown in C1 were traced with Simple Neurite Tracer plug-in followed by process ‘filling’ as detailed in Experimental procedures. (C) Representative z-stack images of APP_sw,Ind_ Tg mice showing cells stained for CD68 (Ca), CD39 (Cb), and merge image (Cc). (D) Representative z-stack images of APP_sw,Ind_ Tg mice stained for CD11b (Da), CD39 (Db), and merge image (Dc). (E) Graph showing CD39 mean fluorescent intensity (MFIs) of IbaI^+^ cells in images taken from 3- and 21-month-old WT mice and APP_Sw,Ind_ (aged 9 months), and APP/PS1 (aged 15 months) Tg mice. Average MFIs were calculated for each experimental group and analyzed by a one-way Tukey’s ANOVA. **P < 0.01, ***P < 0.001. Percent of CD39^low^ and CD39^high^ cells among the CD39^+^ population was evaluated based on CD39 fluorescence intensity levels. Lower and upper thresholds (horizontal broken lines) were set according to 3-month-old WT mice. (F) A 3-D image of APP_sw,Ind_ stained for IbaI^+^ cells in the vicinity of Aβ plaques reconstructed with Simple Neurite Tracer plug-in and viewed with 3D Viewer (FIJI software). Three IbaI^+^ cell populations, distinct by their morphology appearance, are depicted in the image: 1st layer of amoeboid cells (yellow); 2nd layer of cells with enlarged cytoplasm and reduced morphological complexity (light blue); and 3rd layer of ramified cells with reduced morphological complexity (white). Counter staining with TO-PRO-3 appears in dark blue.

As microglial process complexity is significantly affected in the cerebral cortex of APP Tg mice, we asked whether the molecular milieu at the plaque surrounding impacts on process properties. We thus chose to analyze microglial co-expression of activation markers and CD39 (also known as ectonucleoside triphosphate diphosphohydrolase 1), which plays a key role in microglial migration, process formation toward ATP and phagocytosis (Davalos *et al*., 2005; Farber *et al*., 2008; Sieger *et al*., 2012; Bulavina *et al*., 2013). Co-expression of CD39 by IbaI+ cells was evaluated in young (3 months old) and old (21 months old) WT mice, APP_Sw,Ind_ Tg mice (9 months old), and APP/PS1 Tg mice (15 months old). Figure 5A,B shows that compared with the majority of microglial cells in WT mice, which are IbaI^+^/CD39^+^, plaque-associated microglia in APP_Sw,Ind_ Tg mice express high levels of CD39 (CD39^high^, Fig. 5B,C1). However, in the plaque surrounding, microglia are detected as either CD39^+^ (Fig. 5B,C2) or CD39^low^ (Fig. 5B,C3). Co-immunostaining of CD39 with either CD68 (Fig. 5C) or CD11b (Fig. 5D) shows that CD39^high^ cells are mostly found in the vicinity of Aβ plaques and express high levels of CD68 and CD11b. Mean fluorescent intensity (MFI) of Alexa-546-conjugated anti-CD39, calculated for individual IbaI+ cells in the cortex (layer 2–3, 0 to −2 mm lateral to bregma), shows that while the average CD39 MFI in young WT mice did not differ from old WT mice (617.2 ± 8.2 and 644.7 ± 11.3 in young and old mice, respectively), it was significantly increased in APP_Sw,Ind_ Tg mice (673.7 ± 14.1; *P* < 0.001) and to a further extent in APP/PS1 Tg mice (729.7 ± 9.8; *P* < 0.001 compared with young mice, and *P* < 0.01 compared with old mice). The prevalence of CD39^high^ and CD39^low^ cells among IbaI^+^ cells (see thresholds set to WT populations) was specifically increased in APP_Sw,Ind_ and APP/PS1 Tg mice (Fig. 5E).

Overall, three layers of cells accumulate in the plaque surrounding: 1st layer: IbaI^+^/CD39^high^ amoeboid cells found in close proximity to the plaque (Fig. 5F, yellow cells); 2nd layer: IbaI^+^/CD39^high^ cells with enlarged cytoplasm and decreased morphological complexity compared with cells found in plaque-free areas (Fig. 5F, light blue cells and Table S2); and 3rd layer: IbaI^+^/CD39^+^ cells with a marked population expressing low levels of CD39 and exhibit decreased morphological complexity compared with cells found in plaque-free areas (Fig.5F, white cells).

**Figure 6:**
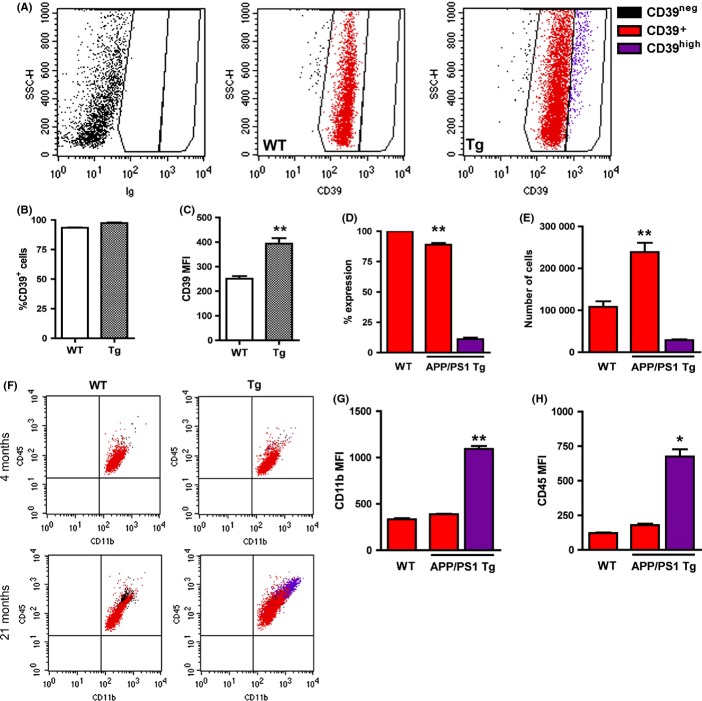
CD39^high^ cells in amyloid precursor proteins (APP)/PS1 Tg mice have increased expression of CD11b and CD45. (A) Flow cytometry profiles showing CD39 expression by CD11b^+^CD45^+^ gated cells in 21-month-old WT and APP/PS1 Tg mice. Cell populations were defined based on fluorescence levels in isotype-stained control (left panel) and identified throughout the figure as CD39^−^ (black), CD39^+^ (red), and CD39^high^ (purple). Quantification of FACS data showing %CD39^+^ among CD11b^+^CD45^+^ cells (B) and CD39 mean fluorescent intensity (MFI) (C) in WT and APP/PS1 Tg mice. (D) Percent CD39^high^ cells among CD39^+^ cells. (E) Numbers of CD39^+^ and CD39^high^ CD11b^+^CD45^+^ cells in WT and APP/PS1 Tg mice. (F) CD45/CD11b plots from WT and APP/PS1 Tg mice at 4 and 21 months of age, with CD39 subpopulations identified by multicolor gating, as defined above. CD11b (G) and CD45 (H) MFIs among CD39^+^ cells in WT and APP/PS1 Tg mice. *P < 0.05, **P < 0.01.

To further characterize the distribution and phenotype of CD39^+^ subpopulations in APP Tg mice, we performed FACS analysis of microglia from the cortex of 21-month-old WT and APP/PS1 Tg mice. Figure 6A–B shows that 93% and 97% of CD45^+^CD11b^+^ cells were CD39^+^ in WT and Tg mice, respectively, and that the MFI of CD39 was significantly higher in APP/PS1 Tg mice than WT mice (Fig. 6C). Approximately 15% of the CD39^+^ cells in APP/PS1 Tg mice expressed significantly higher levels of CD39 (CD39^high^) (Fig. 6D,E). CD45^high^CD11b^high^CD39^high^ cells were essentially absent in young and old WT (Fig. 6F, left panels) and in young APP/PS1 Tg mice (Fig. 6F, upper right panel) but occurred with significant numbers in APP/PS1 Tg mice (Fig. 6F–H). Taking together, whereas the CD39^low^ cells detected with IHC analysis were not detected by FACS analysis, CD39^high^ cells expressing high levels of CD11b and CD45 appeared as a distinct population of activated microglia presumably representing the plaque-associated microglia we observed in tissue sections.

## Discussion

We hereby provide an accurate quantitative technique for morphometric analysis of individual microglia based on 3-D reconstruction of the cells. Utilizing this technique, we show that microglial fine processes are significantly reduced in old mice, and that overexpression of the human mutated APP and the deposition of plaques in the brain significantly accelerate this phenomenon. Our results thus demonstrate a considerable age-dependent decrease in the scanning capacity of microglia, which is additionally reduced in mouse models of AD at least in part due to microglia accumulation around Aβ plaques. Microglia stratified in all three layers around Aβ plaques had distinct morphology and process characteristics compared with ramified resting microglia in the healthy CNS.

The variety of receptors that microglia express, their ramified morphology, and their dynamic nature allow them to continuously survey their environment to maintain and repair the brain. Whereas morphological analyses are widely implemented to study neurons and astrocytes (Matyash & Kettenmann, 2010; Saijo & Glass, 2011), only a few studies have utilized these morphometric methods to characterize microglial morphology and how it changes after various inflammatory triggers (Damani *et al*., 2011; Fontainhas *et al*., 2011). In the current study, we first assessed and characterized the ramification properties of cortical microglia in brains of healthy mice. This was accomplished by 3-D digital tracing of individual microglia, either using GFP expressed under the CX3CR1 promoter or using immunohistochemical analysis of IbaI, as both CX3CR1 and IbaI are expressed abundantly in microglia (Kanazawa *et al*., 2002). A similar morphometric analysis was recently performed for retinal microglia, using a 2-D analysis based on skeletonization of confocal z-stack images (Damani *et al*., 2011). Our study required 3-D analysis, because microglial cells in the cortex gray matter showed greater overlap of processes (reflected by their increased branch length and tree area).

Our study demonstrates that cortical microglia show three orders of process hierarchy: (i) approximately 7–9 main processes extending directly from the cell soma; (ii) medium processes branching from the main processes to several shorter and thinner processes; and (iii) very fine processes, which are mostly devoid of IbaI expression. Quantitative analysis of process complexity revealed that cortical microglia in aged mice showed a significant reduction in the number and total length of their processes. The reduction in microglial fine processes with age was less pronounced when the analysis was based on IbaI staining. We believe this is primarily because IbaI is not evenly dispersed in the cytoplasm and is mainly present in the ‘backbone’ of microglia, rather than in their fine tips. Similar to previous observations in humans (Streit & Xue, 2009), IbaI staining in our study appeared fragmented, presumably reflecting the functional role of IbaI in membrane ruffling and phagocytosis (Kanazawa *et al*., 2002). We therefore speculate that the more robust decline in microglial processes that were observed in GFP-labeled microglia compared with IbaI-stained microglia was due the even distribution of GFP (expressed under the CX3CR1 promoter) throughout the cell and its detection at the very fine processes.

Whereas a significant decline in fine processes of cortical microglia was observed at 21 months of age, a similar ‘old’ phenotype was observed already at 9–15 months of age in mouse models of AD. Except for the tree area, the branching, bifurcations, and total branch length were reduced in this AD-like mouse model to levels similar to those observed in old WT mice, namely at 21 months of age. In addition, Sholl’s analysis and grid analysis revealed a significant reduction in microglial processes surrounding the plaque. Such compromised morphology of microglia was not observed in young APP Tg mice before Aβ plaques are generated suggesting that the ‘old’ phenotype observed in adult APP Tg mice is due the accumulation of Aβ in the brain.

A closer look at the plaque area revealed three layers of microglia distinct from WT resting microglia: amoeboid (1st layer, CD39^high^), semi-amoeboid (2nd layer, CD39^high^), and ramified (3rd layer, CD39^+^ and CD39^low^) (see Fig. 5E). Whereas the 1st and 2nd layers contained activated CD45^high^/CD11b^high^/CD68^high^ microglia, the outer 3rd layer contained ramified microglial cells expressing lower levels of CD39. Microglia are the only cells identified so far in the brain parenchyma that express CD39 (Braun *et al*., 2000) and thus effectively hydrolyze ATP and ADP to AMP (Bulavina *et al*., 2013). Interestingly, such degrading capacity of ATP appears to play a key role in microglia migration (Farber *et al*., 2008; Sieger *et al*., 2012), phagocytosis (Bulavina *et al*., 2013), and process formation (Braun *et al*., 2000). However, whereas hydrolyzing ATP by CD39 on microglia plays a key role in ATP-induced migration, it attenuates the P2Y6 receptor-mediated phagocytic activity of microglia (Farber *et al*., 2008; Bulavina *et al*., 2013). The increased levels of CD39 in Aβ plaque-associated microglia may thus reflect their highly migration capacity to the plaques on one hand, and their reduced capacity of Aβ uptake on the other. In addition, because microglia in CD39 KO mice exhibit significantly reduced microglial coverage (Braun *et al*., 2000), the reduced levels of CD39 on microglia surrounding the plaques may, at least in part, explain their compromised morphology. By hydrolyzing ATP and ADP at sites of Aβ plaques, CD39 may thus play a key role in regulating microglial migration, process formation, and phagocytosis. Both the expression dynamics and function of CD39 in microglia should be further investigated in the context of AD pathology.

One important question is whether such distribution of abnormal phenotypes of microglia around Aβ plaques contributes to cognitive decline in mouse models of AD. Cognitive decline indeed appears to result from brain inflammation which occurs with aging (Villeda *et al*., 2011; Weinstock *et al*., 2011) or with peripheral inflammation, for example, upon intraperitoneal injection of lipopolysaccharide (LPS) (Corona *et al*., 2012; Cunningham, 2013). Such inflammatory reactions indeed result in thicker and less branched and motile microglial processes which overall exhibit reduced coverage area (Cunningham, 2013). Microglial accumulation near Aβ plaques may thus not only shift the molecular and cellular milieu to one that can enhance neurotoxicity (Varvel *et al*., 2012; Heneka *et al*., 2013), but also cause a progressive decrease in microglial process complexity that may impair the clearance of Aβ oligomers, modulation of the synaptic network, and/or neuronal repair processes. A recent study has elegantly demonstrated that microglia depletion in adult mice results not only in general cognitive deficits but also in reduced motor activity-dependent synapse formation within the motor cortex (Parkhurst *et al*., 2013). The authors further showed that such microglial activity presumably results from their ability to regulate synaptic proteins via local production of brain-derived neurotrophic factor (BDNF) (Parkhurst *et al*., 2013). Given the growing body of evidences showing that morphological changes in Aβ plaque-associated microglia are not readily translated to a robust upregulation of proinflammatory cytokines (Gomez-Nicola *et al*., 2013) and that the amount of Aβ soluble species correlates with cognitive decline better than the amounts of plaques (Harris *et al*., 2010; Jin *et al*., 2011), it is plausible that the remarkable decrease in microglial process complexity associated with Aβ plaques is a significant factor in the accelerated cognitive decline observed in mouse models of AD.

The removal of Aβ from the brain, for example, by Aβ antibodies (Morgan *et al*., 2000; Hock *et al*., 2003; Cramer *et al*., 2012), may thus not only clear synaptotoxic forms of Aβ but also reduce neurotoxic inflammation and microglial dysfunction by restoring microglial coverage. Such a therapeutic approach may, however, be more efficient if conducted in a prevention mode and combined with an immunomodulating agent that shapes the inflammatory reaction that occurs in the brain with aging.

## Experimental procedures

### Mice

C57BL6 mice were purchased from Harlan, Israel. APP_Sw,Ind_ Tg mice (line J20) (Mucke *et al*., 2000) on a C57BL6 background expressing APP under the platelet-derived growth factor promoter (PDGF) were kindly provided by Prof. Mucke. APP_Sw_/PS1-dE9 Tg mice (APP/PS1) (Jankowsky *et al*., 2004) were purchased from Jackson Laboratory (Bar Harbor, ME, USA). WT and APP/PS1 Tg mice used in flow cytometry experiments were bred and housed at the animal facility at the University of Southern Denmark. C57BL6 CX3CR1^+/GFP^ mice that harbor a targeted replacement of a single copy of the CX3CR1 gene by a GFP-reporter gene were kindly provided by Prof. Jung (Jung *et al*. 2000). For all experiments, similar numbers of female and male mice were used. All surgical and experimental procedures were approved by the Institutional Animal Care and Use Committee (IACUC) of Ben-Gurion University of the Negev, Israel (Approval Number IL-610904) or under permission from the National Danish Animal Research Committee (2011/561-1950).

### Immunohistochemistry

Animals were deeply anesthetized with an overdose of isoflurane and were perfused cardially with 20 mL of PBS. Brains were then excised and incubated in 4% paraformaldehyde (PFA) overnight and transferred to 30% sucrose solution for 48 h at 4 °C. The tissues were frozen and stored at −80 °C. Immunohistochemical staining was performed on free-floating 50-μm sections cut in a cryostat. For IbaI, CD11b, and CD39 staining, sections were washed with PBS and then incubated with PBS with 0.05% triton. The sections were then incubated in primary antibody diluting buffer (Golden Bridge International, Mukilteo, WA, USA) and then transferred for overnight incubation with IbaI antibody (WAKO, Osaka, Japan), CD39 (R&D, Minneapolis, MN, USA), and CD11b (Serotec, Raleigh, NC, USA) at 4 °C. The sections were then washed with PBS and incubated for 1 h with AlexaFluor 488 or 546 goat anti-rabbit (Invitrogen, Carlsbad, CA, USA) for IbaI staining and anti-sheep NL557 (R&D) for CD39 staining. Where indicated, slices were counterstained with TO-PRO-3 iodide (642/661) (Invitrogen).

### Confocal microscopy

Confocal microscopy was used to capture fluorescently labeled cells in slices. All images were obtained using the Olympus FluoView 1000 confocal microscope (Olympus, Hamburg, Germany) at a 1024 × 1024 pixel resolution with ×60 objective. For reconstruction of microglial cells, z-stack images were taken through 20–40 μm thickness at 0.5-μm intervals. For the modified Sholl’s analysis, images were taken through 20 μm thickness (excluding the upper and lower edges of the tissue) at 1-μm intervals.

### Image analysis

#### 3-D reconstruction of individual microglial cells

Z-stack confocal images of 20–40 μm thickness at intervals of 0.5 μm were taken at layers 2/3 of the cortex (0 to −2 mm lateral to bregma). The images were reconstructed with the Simple Neurite Tracer plug-in bundled in FIJI software (freely downloadable from http://fiji.sc/Fiji). Manual tracing of each process along the stack was achieved by selecting points along the midline of a process while the software finds a path between them. For volume reconstruction, we used the ‘filling’ option based on a defined threshold. The filler option explores the image starting from the path until it reaches an intensity threshold set by the user. The resulting z-stack images were transferred for visualization with either 3D Viewer of the FIJI software or with Volocity software (PerkinElmer, Waltham, MA, USA), as indicated.

#### Morphometric parameters calculation

The following morphometric parameters were calculated with L-measure software (Scorcioni *et al*., 2008): (i) total branch length; (ii) number of bifurcations; (iii) number of branches. After tracing an individual microglial cell using FIJI software, the corresponding file was transferred to the L-measure software. For analysis, only branches longer than 0.5 μm were counted. The calculated parameters were then extracted for later analysis with Microsoft Excel. To compare between WT and APP mice, all parameters were normalized for 20 μm thickness.

#### Coverage volume

Coverage volume is presented as the length of branches within a 10 μm^3^ unit of tissue volume. This parameter was calculated by dividing the total branch length of individual microglia by its volume. The volume of individual cells was calculated by multiplying the tree area value by the thickness of the microglia, as revealed by the tracing analysis. The resulting length was multiplied by 1000 to estimate the length within a 10 μm^3^ unit volume.

#### Spatial coverage of microglia: modified Sholl’s analysis and Grid analysis

The following groups were analyzed: (i) C57BL6 mice at 3, 9 and 21 months of age; (ii) APP_Sw,Ind_ mice at 3 and 9 months of age; and (iii) APP_Sw_/PS1-dE9 Tg mice at 15 months of age (these mice were excluded from the modified Sholl’s analysis). Brain sections from these mice were stained with IbaI and the cortex (0 to −2 mm lateral to bregma) imaged with confocal microscopy at ×40 magnification through 20-μm-thick slices at 1-μm intervals. For APP_Sw,Ind_ mice, only plaques with an average of 40 μm radius were imaged. Thereafter, gray-level maximum z-projection images were set to eliminate background based on intensity threshold. These images were converted to binary images, processed with the ‘skeletonize’ option in FIJI software, and further analyzed with a modified Sholl’s analysis and grid analysis. A representative heat-map image was generated based on 8-bit z-projection image using the 3D Surface Plot plug-in bundled in FIJI software.

#### Grid analysis

Grid analysis was performed by placing a 32*32 grid (each square 25 μm^2^) on top of the z-projection skeletonized image and measuring the total length of processes in each square of the grid. These results were either summed for each group and presented as a distribution histogram with binning of 1 μm, or averaged and presented by bar graphs.

#### Modified Sholl’s analysis

To calculate the number of intersections between microglial processes and concentric circles originated from the center of the plaque (for APP_Sw,Ind_ mice) or from an individual arbitrary cell (for WT mice), we used the Sholl’s analysis plug-in (Ghosh Lab, UCSD, San Diego, CA, USA) bundled in FIJI. This was used to analyze the number of intersections in circles with a radius between 40 μm (minimal distance from the center of the plaque) and 140 μm (the average distance to the neighboring plaque, excluding the edges of the image) with a step size of 5 μm. To quantify the spatial coverage of microglia, we modified the conventional Sholl’s analysis by normalizing the number of intersections to the perimeter enclosed by its circle. The results were then presented as the number of intersections along 1 mm of an arbitrary line.

#### Mean fluorescent intensity

Brain sections were immunolabeled with rabbit anti-mouse IbaI and sheep anti-mouse CD39 followed by secondary staining with Alexa Fluor® 488-conjugated goat anti-rabbit and Alexa Fluor® 546-conjugated goat anti-rat (Invitrogen, Grand Island, NY, USA). 20-μm-thick z-stack images with 1-μm interval were then taken with a confocal microscope, and MFIs were calculated using the Volocity software. Identical imaging settings were used for all samples. Intensity threshold was first set to identify IbaI+ cells in the image. CD39 MFI of each individual IbaI cell was then obtained by Volocity software. To define CD39^low^ and CD39^high^ cells, we set lower and upper thresholds according to the distribution of CD39 fluorescence intensity in 3-month-old WT mice. The percentage of cells below the lower threshold (CD39^low^) or higher than the maximum threshold (CD39^high^) was calculated out of the total numbers of IbaI+ cells in each experimental group.

### Quantitative PCR

Mice were perfused with PBS, and half brains were immediately frozen in liquid nitrogen and stored in −80 °C. RNA was extracted by a phenol–chloroform procedure and analyzed with Bioanalyzer (Agilent Technologies, Santa Clara, CA, USA). 2 μg of RNA was reverse transcribed with high-capacity cDNA reverse transcription kit (Applied Biosystems, Invitrogen, CA, USA). One hundred ng of cDNA was used per 20 μL of TaqMan real-time PCR reaction (Invitrogen). Samples were run in triplicates. The Cxxc1 gene was used as an endogenous control to normalize gene expressions.

### Flow cytometry

After PBS perfusion, brains were carefully removed, stripped of meninges, and the neocortex from each mouse was isolated and processed separately. This tissue was homogenized through a 70 μm cell strainer, blocked for nonspecific binding, and stained for surface markers (Babcock *et al*., 2006). The antibodies used were PE-conjugated rat anti-mouse CD45 (clone 30-F11; BD Biosciences), PerCPCy5.5-conjugated rat anti-mouse CD11b (clone M1/70; BD Biosciences, San Jose, CA, USA), and eFluor® 660-conjugated rat anti-mouse CD39 (clone 24DMS1; ebioscience, San Diego, CA, USA), as well as isotype-matched controls. Equivalent fractions of cells from the neocortex were stained, similar numbers of total events were collected before gating, and estimates of cell numbers were generated as previously described (Babcock *et al*., 2006). CD11b^+^ CD45^+^ cells were identified using a series of gates [forward scatter (FSC) vs. CD11b, side scatter (SSC) vs. CD11b, FSC vs. CD45 and SSC vs. CD45]. Thereafter, CD39 subpopulations were defined in SSC vs. CD39 plots based on fluorescence levels in isotype-stained controls and level of expression in WT mice.

### Statistics

Statistical significance was tested using t-tests (Prism software), unless stated otherwise. Graphs indicate mean ± SEM, with n representing the number of mice or slices, as indicated. Comparisons among three or more data sets were performed using a one-way analyses of variance (ANOVA) with the Tukey’s comparison post hoc test for Gaussian distributions. For quantitative PCR, ANOVA was adjusted by Benjamini–Hochberg false discovery rate. Two-tailed paired t-tests (Prism) were used for comparison of CD39^low^ and CD39^high^ populations within APP/PS1 Tg mice by flow cytometry.

## Supporting Information

**Fig. S1** IbaI-stained microglial process complexity deteriorates with aging.

**Fig. S2** Microglia accumulation at sites of Aβ deposition in mouse models of AD.

**Table S1** Microglial process complexity deteriorates with aging.

**Table S2** Morphological analysis of IbaI+ cells close and distant from Aβ plaques in APPsw, Ind.
