# Supplementary figures and images for: Accelerated microglial pathology is associated with Aβ plaques in mouse models of Alzheimer’s disease
Source: Aging Cell. 2014 Mar 18;13(4):584–95. doi: 10.1111/acel.12210 (PMC4326940; doi:10.1111/acel.12210)

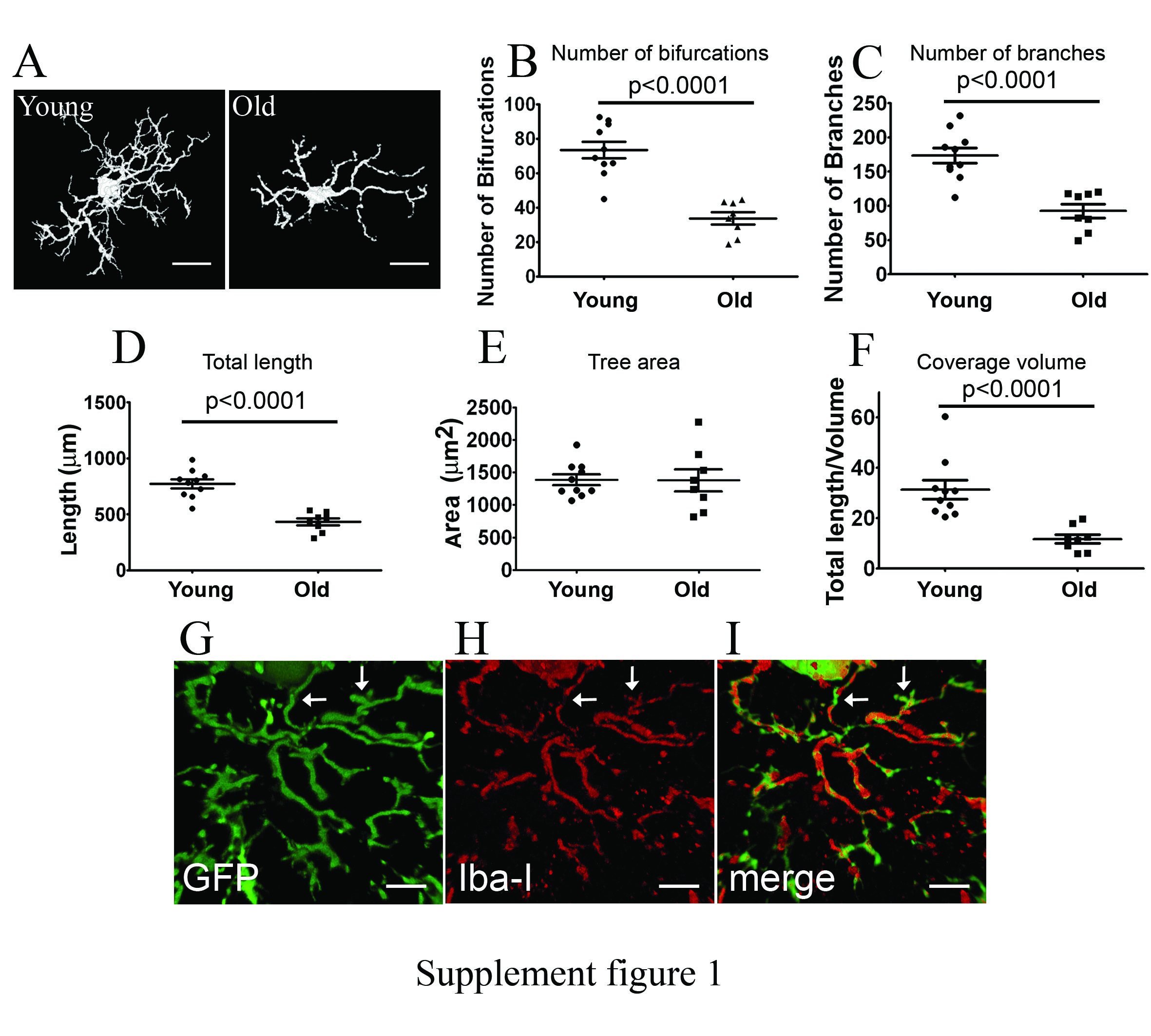

Supplement: Supplementary file 1 — Fig. S1 IbaI-stained microglial process complexity deteriorates with aging. [file acel0013-0584-sd1.jpg]

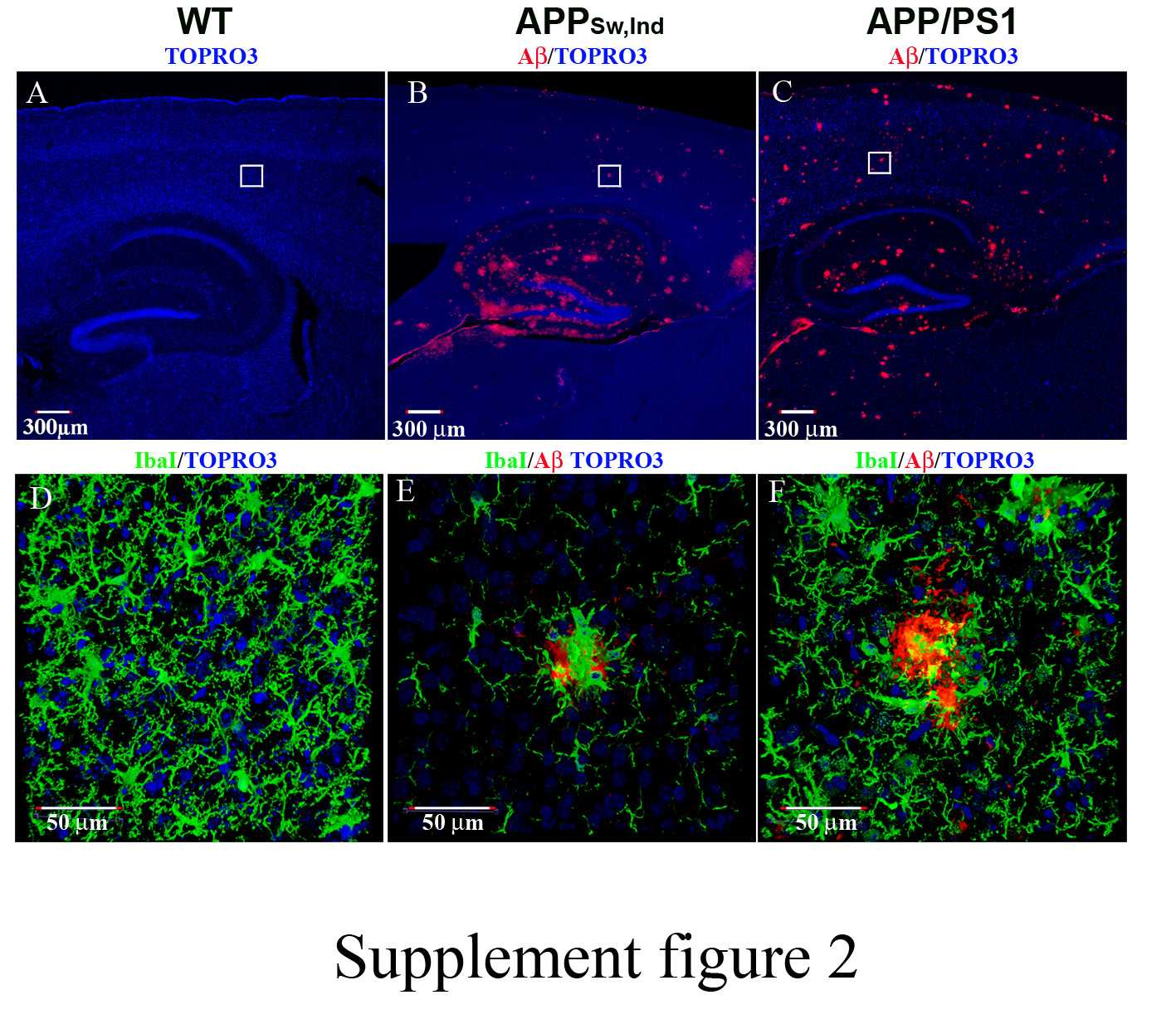

Supplement: Supplementary file 2 — Fig. S2 Microglia accumulation at sites of Aβ deposition in mouse models of AD. [file acel0013-0584-sd2.jpg]

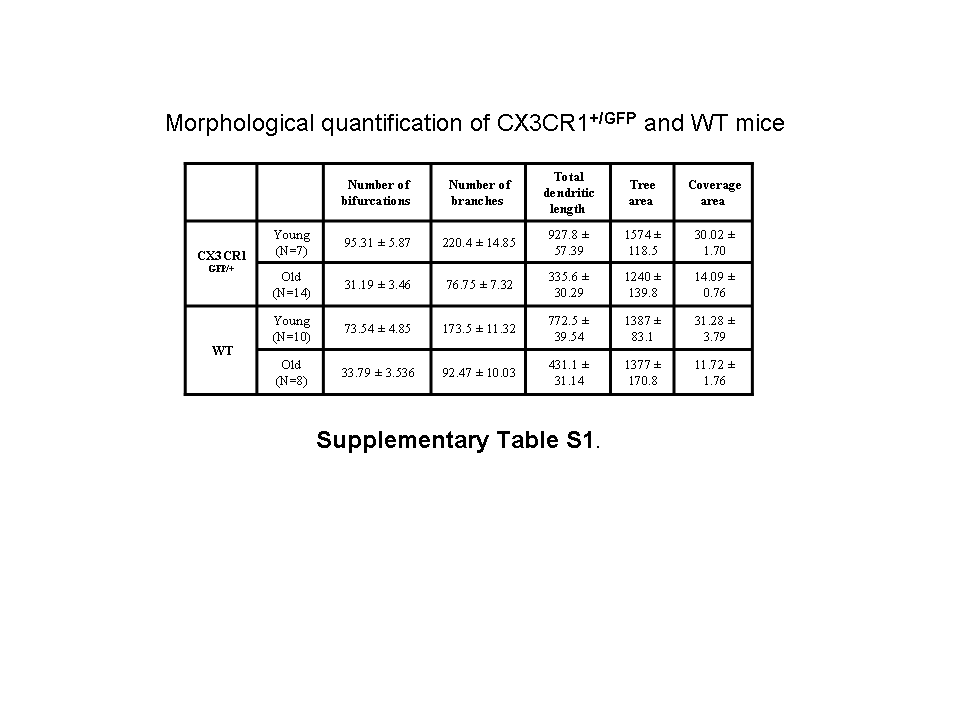

Supplement: Supplementary file 3 — Table S1 Microglial process complexity deteriorates with aging. [file acel0013-0584-sd3.tif]
