# Supplementary material for: Accelerated microglial pathology is associated with Aβ plaques in mouse models of Alzheimer’s disease
Source: Aging Cell. 2014 Mar 18;13(4):584–95. doi: 10.1111/acel.12210 (PMC4326940; doi:10.1111/acel.12210)
Supplement: Supplementary file 4 — Table S2 Morphological analysis of IbaI+ cells close and distant from Aβ plaques in APPsw, Ind. [file acel0013-0584-sd4.ppt]

## Slide 1
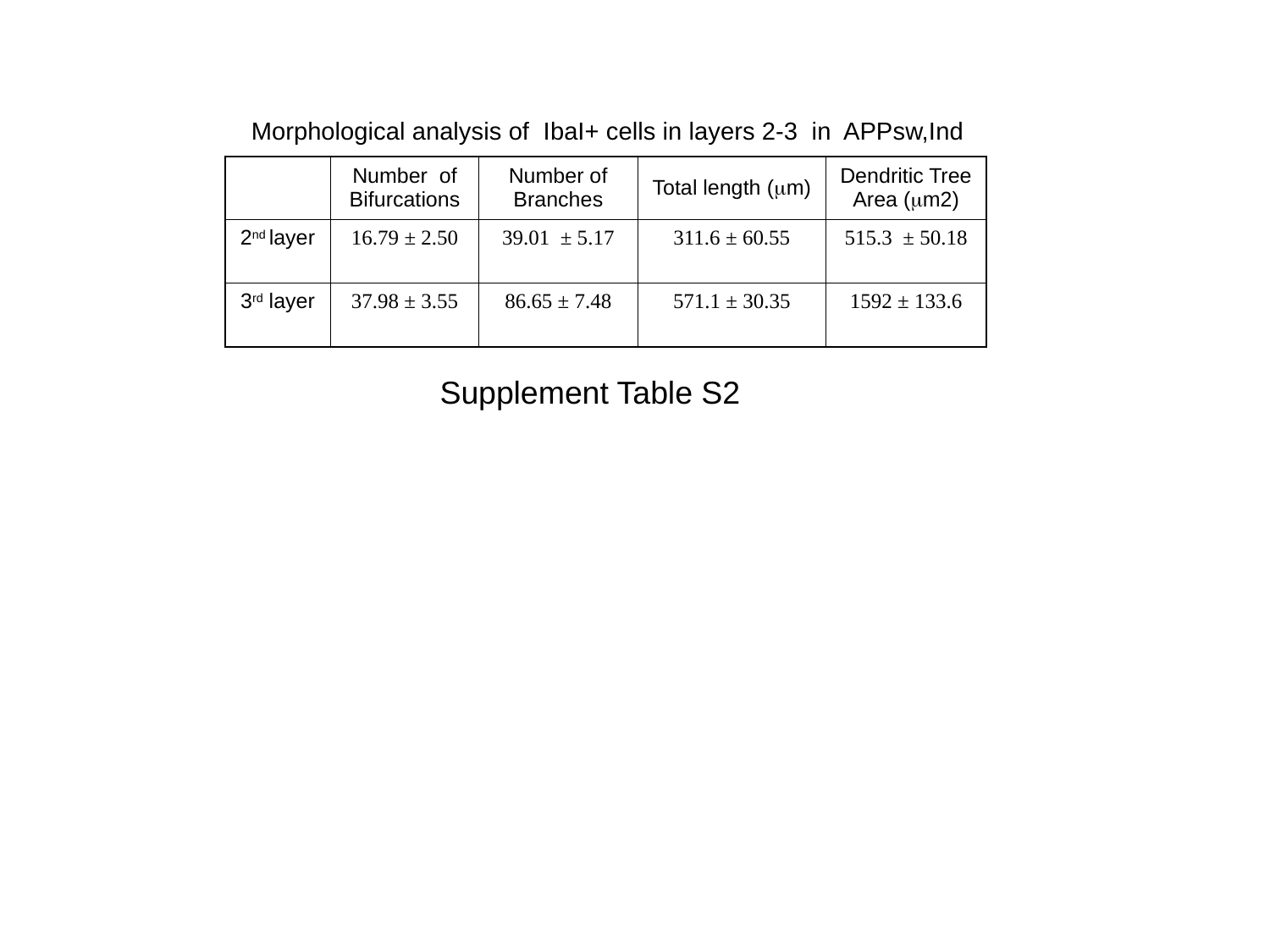

Morphological analysis of IbaI+ cells in layers 2-3 in APPsw,Ind
| | Number of Bifurcations | Number of Branches | Total length (μm) | Dendritic Tree Area (μm2) |
| --- | --- | --- | --- | --- |
| 2nd layer | 16.79 ± 2.50 | 39.01 ± 5.17 | 311.6 ± 60.55 | 515.3 ± 50.18 |
| 3rd layer | 37.98 ± 3.55 | 86.65 ± 7.48 | 571.1 ± 30.35 | 1592 ± 133.6 |
Supplement Table S2
