# Supplementary material for: Accelerated microglial pathology is associated with Aβ plaques in mouse models of Alzheimer’s disease
Source: Aging Cell. 2014 Mar 18;13(4):584–95. doi: 10.1111/acel.12210 (PMC4326940; doi:10.1111/acel.12210)
Supplement: Supplementary file 5 [file acel0013-0584-sd5.docx]

**Supplementary Figure 1. IbaI-stained microglial process complexity deteriorates with aging.** Brains from young (3 mice; n=10 cells) and old (3 mice; n=8 cells) WT mice were sectioned and immnuolabeled with anti-IbaI. A quantitative analysis of microglial morphology was then performed and is presented as described in previous figures. (A) Representative images of IbaI-labeled microglia from young (left panel) and old (right panel) WT mice, defined by the Simple Neurite Tracer plugin. (B-F) A quantitative analysis of the number of bifurcations (B), the number of branches (C), the total branch length (D), the total area (E) and the coverage volume governed by an individual microglia cell (F). (G-I) A representative Z-projection image of a brain section from CX3CR1^GFP/+^ Tg mice (G) immunolabeled with anti-IbaI (H), and the merged image (I). Arrows indicate (1) processes which were homogeneously labeled with GFP but were not stained with anti-IbaI and (2) fine processes observed with GFP but not with IbaI staining. Bar represents 20μm (A); 5μm (G-I). Of note, two main differences between GFP-labeled (Fig. 1) and IbaI-labeled microglia: (1) IbaI was less abundant in the fine processes (Supplementary Fig. 1I, see arrows); and (2) several processes appeared fragmented when stained with anti-IbaI (Supplementary Fig. 1H, see arrows) but not when imaged for GFP (Supplementary Fig. 1G). These data indicate that IbaI is non-uniformly dispersed in the microglia cytoplasm, but rather in a pattern presumably reflecting its functional dynamics.

**Supplementary Figure 2. Microglia accumulation at sites of Aβ deposition in mouse models of AD.** Brain sections derived from representative WT (left panel), APPsw,Ind (middle panel) and APP/PS1 (right panel) Tg mice were immunolabeld with anti-Aβ (red) and IbaI (green). Z-projection images of WT (A), APPsw,Ind (B) and APP/PS1 (C) Tg mice. (D-F) Higher magnification of the area denoted by a white rectangle in (A,B).

**Table S1**. **Microglial process complexity deteriorates with aging.** Microglial morphology in the cortex (layers 2/3) was assessed in young versus old CX3CR1^GFP/+^ Tg mice and WT mice immunolabled with anti-IbaI as described in Figures 2 and 3. Quantitative analysis of the microglial processes (layer 2/3) was preformed with the L-measure software and statistically analyzed with Graph Pad. The table represents the corresponding mean ± SEM for graphs which appear in Figs. 2-3.

**Table S2.** **Morphological analysis of IbaI+ cells close and distant from Aβ plaques in APPsw,Ind.** Microglial morphology based on IbaI staining was assessed in APPsw,Ind Tg mice as described in Expermental Procedures. Quantitative analysis of the microglial processes was preformed with the L-measure software. The table represents the corresponding mean ± SEM for IbaI^+^ cells in layer 2, 3 as shown in Fig. 5E.
